# Nutrient intake and renal cancer: molecular pathways and mechanistic insights into the protective role of dietary components

**DOI:** 10.3389/fnut.2026.1762070

**Published:** 2026-02-26

**Authors:** Peng Chen, Xiaojun Bi, Renli Tian, Qian Zhang

**Affiliations:** Department of Urology, General Hospital of Northern Theater Command, Shenyang, China

**Keywords:** dietary fiber, metabolic reprogramming, molecular pathways, nutrient intake, nutrigenomics, renal cancer, short-chain fatty acids (SCFAs)

## Abstract

Renal cell carcinoma involves specialized metabolic transformations centered on proximal tubule biology, yet its interface with nutrient intake is frequently interpreted within a generalized oncological framework. This review contextualizes dietary influences within the kidney-specific physiological environment, emphasizing the role of renal filtration dynamics and oxygen-sensing mechanisms in shaping nutrient–tumor interactions. We discuss mechanistic and experimental evidence suggesting that dietary components—particularly fermentable fibers and plant-derived phytochemicals—may function as context-dependent biochemical modulators within the renal microenvironment. Special attention is given to short-chain fatty acids generated by gut microbial fermentation, which may act as distal modulators along the gut–kidney axis and influence metabolic and inflammatory signaling relevant to renal carcinogenesis. By relating circulating nutritional metabolites to proximal tubule metabolic sensitivity and VHL–HIF–dependent regulation, this review aims to bridge systemic nutritional metabolism with metabolic reprogramming characteristic of kidney cancer. Overall, this kidney-centric perspective reframes nutrition from a broad health factor to a context-dependent molecular modulator within renal metabolic pathways, specifically identifying nutritional signals as biochemical modulators—such as short-chain fatty acids—that directly interface with the oncogenic microenvironment through the VHL-HIF and mTOR circuits.

## Graphical abstract

**Figure:**
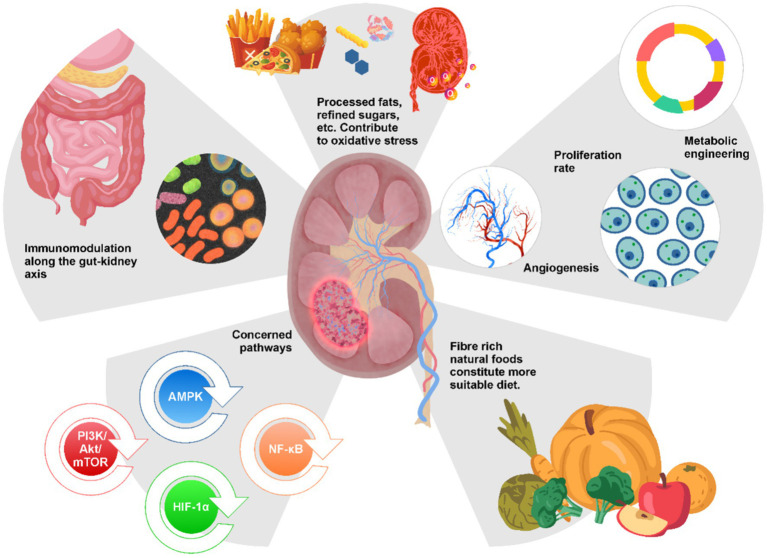

## 1 Introduction to renal cancer and dietary influence

Cancer of the kidneys is an intensely metabolic malignancy characterized by profound nutrient-dependent reprogramming. This metabolic plasticity facilitates distinct genetic diversity and contributes to its high degree of resistance to systemic treatments. Renal cancer incidence is increasing globally due to an increase in the incidence of metabolic disorders (such as obesity and hypertension) and poor eating habits. Renal cancer generally develops from the epithelial cells lining the renal tubules and often progresses slowly before reaching advanced stages, thus, prevention of renal cancer development is best achieved through modification of modifiable risk factors (such as diet) rather than waiting for renal cancer to develop into a late stage (1). Furthermore, while dietary composition is a primary focus, it is vital to acknowledge that other lifestyle factors, including consistent physical activity, sleep hygiene, and psychological stress, independently modulate systemic metabolism and immune surveillance. These components often act as confounding variables that interface with nutritional signals to shape the renal microenvironment.

Nutrition plays an important role in the environment and genetics of the individual who develops renal cancer and, as well, the metabolic sensitivity of the kidney makes nutrition a likely candidate to have a beneficial effect in either promoting or preventing cancer. The renal cortex is metabolically active, and continually metabolizes dietary components and exogenous substances (xenobiotics), and endogenous byproducts. Continued long term consumption of detrimental components in foods (like processed fats, refined sugars, and excessive sodium) will contribute to continued oxidative stress, inflammatory responses, and mitochondrial dysfunction, all of which will contribute to initiation and progression of cancers. However, diets high in fiber, antioxidants, and bioactive phytochemicals may reduce these negative effects by altering cellular and molecular processes (2). Nevertheless, the therapeutic application of antioxidants requires nuanced evaluation, as excessive supplementation can represent a ‘double-edged sword’ in cancer contexts. Emerging evidence suggests that while antioxidants protect healthy cells, they can also potentially shield pre-malignant cells from the oxidative stress required for localized apoptosis or the efficiency of certain cytotoxic therapies.

In recent years there has been an increased scientific interest in understanding the mechanisms of interaction between nutrients and genes, and how those interactions may be used to prevent or treat cancer. Nutritional Oncology is a new field of study that seeks to understand how different nutrients interact with different genes and enzymes and how those interactions may affect epigenetic regulation. For example, bioactive compounds can regulate transcriptional programs that affect angiogenesis, apoptosis, and proliferation, all of which are relevant in the development of kidney cancer. Dietary modulation is not limited to just caloric intake, but also includes the molecular specificity of nutrient interactions with oncogenic or tumor suppressor pathways.

Among the various nutrients being studied, dietary fiber has received a great deal of attention for its numerous systemic effects, although many of them are indirect. As previously discussed, fiber and its fermentation products (short chain fatty acids) are recognized today as regulators of the inflammatory response, immune cell function, and oxidative balance. The systemic effects of fiber are important in renal physiology, where chronic inflammation and metabolic stress provide a microenvironment that fosters malignant transformation. Therefore, fiber signals act as direct biochemical modulators that facilitate renal metabolic homeostasis and enhance the anti-tumor immune response via improved gut-kidney communication (3).

The location of the kidneys in the circulation makes them an intersection point of systemic nutrition as well as local responses to circulating metabolite levels. As such, certain nutrients have been shown to initiate or enhance activation of the NF-κB pathway; the PI3K/Akt/mTOR cascade; and HIF pathways. Each of these pathways has central roles in the decision-making processes cells undergo when they experience some form of stress. The ingestion of diets rich in saturated fats but poor in nutrients derived from plants (i.e., fruits and vegetables) may disrupt all three of these signaling pathways. The disruption of these pathways may lead to angiogenesis and genomic instability; whereas diets rich in antioxidants, complex carbohydrates and unsaturated fats may prevent disruptions of these pathways and restore redox states and signal transduction dynamics (4).

To understand these interactions effectively, investigators must look past general anticancer associations and focus on metabolism within the renals unique organizational context. This study examines how the heavy filtration and intense oxygen demand of the renal cortex transform systemic dietary signs into localized regulators of the tumor microenvironment. By correlating gut-derived metabolites to the systemic biology of oxygen sensing and the specific circuitry of VHL, this manuscript identifies plant-sourced nutrients and antioxidant components as localized biochemical modulators that actively interface with the VHL-HIF and mTOR signaling pathways within the specific pathology of kidney cancer.

### 1.1 Pathophysiology and molecular landscape of renal cancer

Renal Cell Cancer is caused by damage to many molecular pathways involved in regulating the normal physiological environment of the renal cell. Renal cancer has been identified as primarily occurring in the form of Clear Cell Renal Carcinoma (ccRCC) in the Proximal Tubular Epithelium of the Kidneys. The Proximal Tubule regulates the environment to ensure tight control of oxygen supply and substance exchange. Therefore, the tumor cells inability to monitor their own genetic integrity, regulate homeostasis of metabolism and adapt to their environment, appears to contribute to the accumulation of effects of tumorigenesis within the renal cortex.

The kidney is uniquely well-suited to experience hypoxic stress, due to its high oxygen demand and rapid metabolic activity, and thus acts as both a stimulus and facilitator of oncogenic transformation (5).

At the genome level, renal cell cancer is defined by mutations in critical tumor suppressor genes and chromatin remodeling complexes. The VHL gene is a hallmark mutation in ccRCC, which normally directs the degradation of HIF-1α and HIF-2α in normoxic conditions. However, when the VHL gene is mutated or deleted, the HIF proteins are stabilized in normoxic conditions, producing a hypoxic-like condition, triggering uncontrolled angiogenesis and metabolic reprogramming of the cell and providing resistance to apoptosis. In addition, the pseudo-hypoxic signaling produced by the HIF proteins allow the cancerous cells to grow and survive in conditions of limited nutrient availability. Also, the pseudo-hypoxic signaling produces pro-angiogenic factors such as VEGF and PDGF that provide for the continued vascularization of the tumor, ultimately leading to the formation of metastases (6).

In addition to the dysfunction of VHL, recurring mutations in genes such as PBRM1, SETD2, and BAP1 disrupt the chromatin structure and epigenetic landscape, thereby disrupting the regulation of transcription and DNA repair. Together, these mutations have altered the metabolic circuitry of the cell to promote glycolytic flux, reduce fatty acid oxidation, increase ROS levels. This creates an oxidative imbalance that generates mutagenic pressure and inflammatory signals in the microenvironment, creating a favorable environment for tumor growth (7).

One of the most prominent molecular pathways involved in regulating cell growth and survival in kidney cancer is the PI3K/Akt/mTOR pathway. Activation of this signaling pathway results in increased protein synthesis, inhibition of autophagy, and activation of angiogenic pathways, all of which contribute to increased resistance of the tumor. Activation of the MAPK/ERK and NF-κB signaling pathways also stimulates cell proliferation and expression of genes that stimulate inflammation. Therefore, the convergence of these pathways creates a feedback loop where metabolic stress, inflammation, and genomic instability mutually support each other (8).

Another characteristic feature of renal cancer is the ability of tumor cells to adapt their metabolism. Like other cancers, kidney tumor cells display a Warburg-like metabolic profile, using aerobic glycolysis regardless of the presence of oxygen. Adaptation of tumor cells to use aerobic glycolysis provides enough biosynthetic precursors for rapid cell division and proliferation. In addition, this metabolic shift alters the redox balance and utilization of nutrients and links cancer metabolism to the nutritional status of an individual. Therefore, nutrients can modulate how tumor cells respond to oxidative stress and emphasize the responsive nature of the renal tumor microenvironment to changes in dietary intake (9).

The tumor microenvironment (TME), which includes the interactions among immune cells, fibroblasts, endothelial cells, and the components of the extracellular matrix, is also very complex in renal cancer. Chronic inflammation, which can be induced by high fat diets or metabolic syndrome, induces the secretion of pro-tumorigenic cytokines, such as IL-6 and TNF-*α*. These cytokines activate transcription factors, such as STAT3 and NF-κB, which sustain inflammation and enable evasion from the host immune response. Nutrient imbalance can either directly affect tumor cells or alter the TME to create an environment that is supportive of cancer progression.

Finally, epigenetic alterations provide another layer of complexity. Renal tumors show abnormal patterns of DNA methylation and histone acetylation. These patterns inhibit tumor suppressor genes and activate oncogenic transcriptional programs. Metabolites derived from nutrients, such as methyl donor derived from folate and short chain fatty acids derived from fiber fermentation, can modulate these epigenetic markers. Therefore, the availability of nutrients indirectly modulates epigenomic reprogramming and, consequently, the expression patterns of genes in the renal tissue (10).

**Figure 1:**
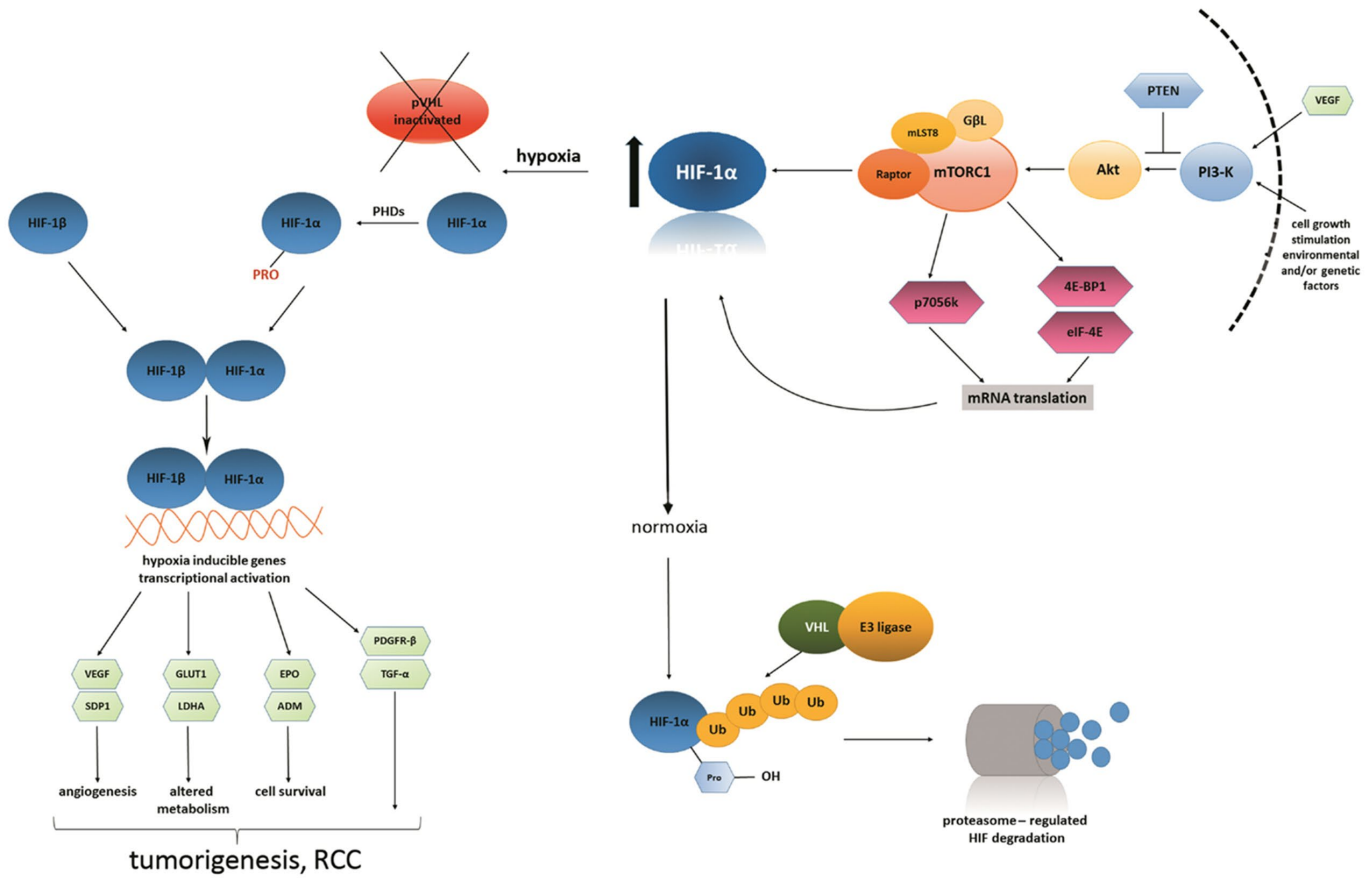
Interconnection between the HIF/VHL and PI3K/Akt/mTOR signaling pathways in renal cancer. Under hypoxia, loss of VHL function appears to contribute to HIF-*α* stabilization and transcription of pro-angiogenic and metabolic genes. Concurrent activation of the PI3K/Akt/mTOR axis amplifies HIF-1α expression, forming a positive feedback loop that promotes angiogenesis, metabolic reprogramming, and tumor progression (13).

Thus, the pathophysiology of renal cancer is a result of the interaction of genetic alterations, metabolic alterations and inflammatory signals. Each molecular pathway is influenced by the nutritional context, therefore establishing diet as a key regulatory factor for susceptibility to oncogenic transformation. Understanding the mechanisms underlying these intersections provides a foundation for developing diet-based prevention and treatment strategies (11, 12). Figure 1 illustrates the relationship between HIF/VHL and PI3K/AKT/mTOR signaling pathways in renal cancer (13).

## 2 Methodology and search strategy

The literature review for this paper utilized a structured approach aimed at obtaining current data linking nutrient intake to renal cancer mechanisms. We systematically searched major scientific databases, including PubMed, Scopus, Web of Science, and Google Scholar, to find peer-reviewed articles, clinical trials, and mechanistic studies published between 2015 and 2025.

Search strings were created using keywords and Boolean operators, including ‘renal cell carcinoma’, ‘nutrient intake’, ‘dietary fiber’, and ‘oncogenic signaling’. To obtain advanced insights, we added additional terms like ‘nutrigenomics’ and ‘gut-kidney axis’. Inclusion criteria mandated that studies concentrate on the nutritional modulation of renal cancer risk or molecular pathways. Conversely, reports lacking mechanistic depth or focusing on non-renal malignancies without mechanistic parallels were excluded.

**Figure 2:**
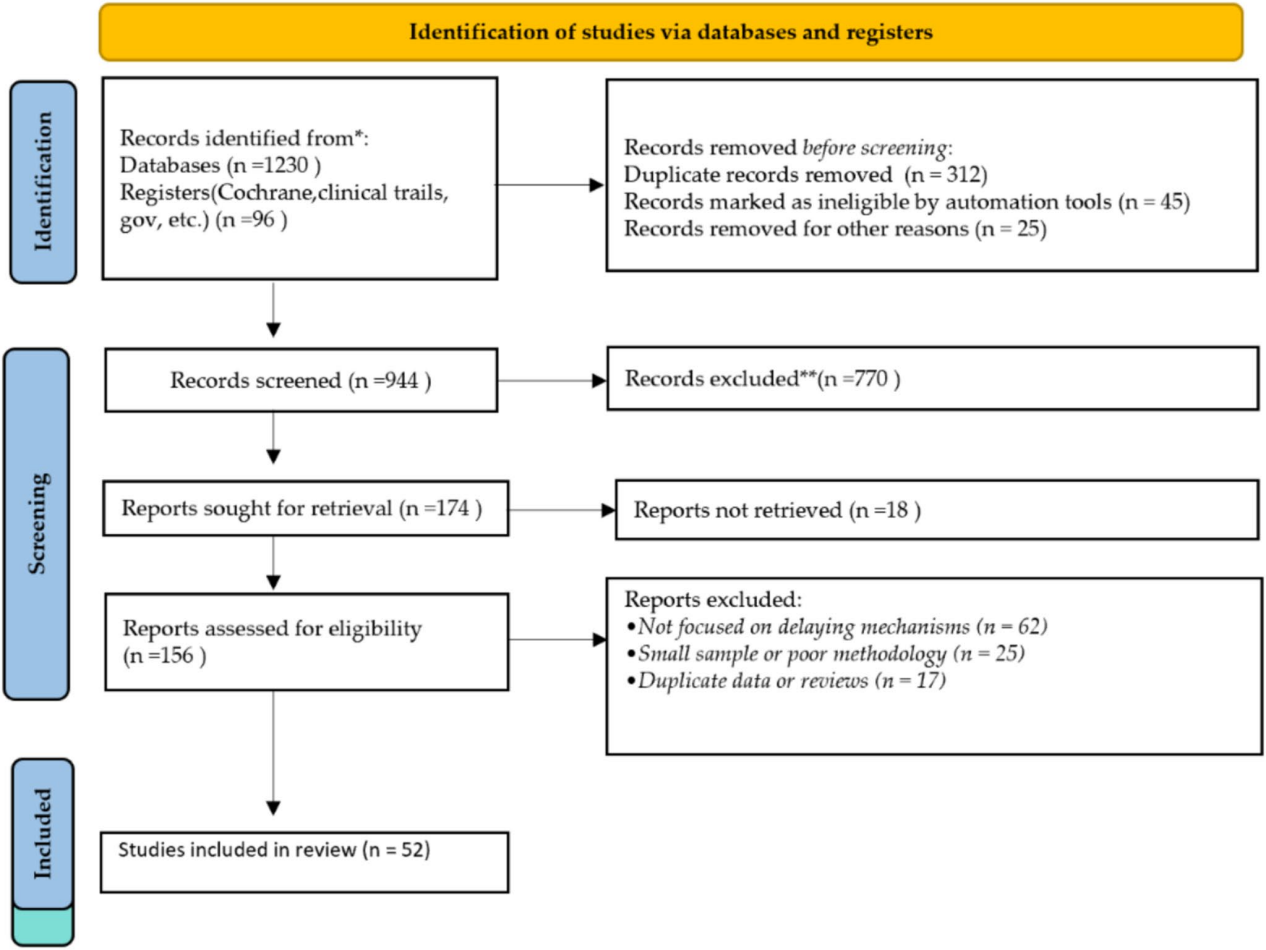
Flow diagram illustrating the literature identification, screening, eligibility assessment, and inclusion process for studies included in this review. A total of 1,326 records were identified through database searching and registry sources. After removal of duplicate records and ineligible studies, 944 records were screened based on titles and abstracts. Of these, 770 records were excluded. Full texts of 174 reports were sought for retrieval, among which 18 reports were not accessible. Subsequently, 156 full-text articles were assessed for eligibility. Reports were excluded due to lack of relevance to mechanistic pathways, insufficient sample size or methodological limitations, or duplication of data. Ultimately, 52 studies were included in the final qualitative synthesis.

The selection process proceeded through systematic identification, screening, and assessing eligibility. The detailed steps of study retrieval and the hierarchy of selection are illustrated in Figure 2. Following these quality appraisal steps, 52 core studies were selected for the qualitative synthesis within this review. These core mechanistic investigations are integrated with current oncology guidelines and physiological background publications to comprise the final analytical body of this work.

## 3 Overview of nutrient classes and cancer modulation

Nutrition is the primary basis of cancer biology; it provides the cell with energy and regulates metabolic and signaling pathways as well as gene expression. All forms of nutrients (carbohydrates, proteins, lipids, vitamins, minerals and phytochemicals) interact with cellular systems in ways that can either promote or suppress the pathways leading to cancer. The sensitivity of renal physiology to the composition of circulating metabolites, due to its complexity and the interdependence of renal physiology and metabolism, creates a strong dependence of renal carcinogenesis on the quality and quantity of nutrient intake (14).

Carbohydrates represent the primary energy sources for human metabolism. However, the carbohydrate-mediated influence on the dynamics of tumors depends upon the type of carbohydrate and the rate of metabolic processing of glucose. High-glycemic diets rich in simple carbohydrates cause increased levels of insulin and insulin-like growth factors (IGF), initiating proliferative cascades such as the PI3K/Akt/mTOR pathway. Hyperglycemia over time generates oxidative stress, endothelial dysfunction and systemic inflammation, all of which promote renal tumorigenesis. Conversely, complex carbohydrates, such as those found in fiber-containing foods, modify the absorption of glucose, reduce plasma insulin levels and generate beneficial metabolites via fermentation. Both processes attenuate metabolic stress that otherwise would activate pro-tumorigenic signaling pathways (15).

Proteins, as another major class of nutrients, perform two distinct functions; they form the structural components of cells and regulate the activity of signaling molecules. Diets rich in animal-derived protein are associated with increased acid–base loads, alterations in renal hemodynamic responses, and elevated levels of nitrogenous waste, all of which can produce renal injury. In addition, certain amino acids, specifically glutamine and serine, serve as metabolic precursors to proliferating cancer cells and provide antioxidant defenses necessary for the synthesis of nucleotides. Therefore, it is critical to maintain a balanced level of protein intake to preserve tissue integrity and immune surveillance. Furthermore, the source and processing of proteins (animal versus plant-based) will determine their contribution to the inflammatory and oxidative states within the renal microenvironment (16).

Finally, lipids are another class of nutrients that have the potential to influence how cancer works. The lipid composition of the diet can directly influence gene transcription, membrane signaling and metabolic reprogramming. Lipid oxidation products formed as a result of consumption of saturated fats and trans-fatty acids will contribute to mitochondrial dysfunction and the generation of pro-inflammatory eicosanoids. Conversely, the consumption of polyunsaturated fatty acids (PUFA), specifically *ω*-3 PUFA, will exert anti-inflammatory effects by limiting the formation of eicosanoids and suppressing the activity of NF-κB. Due to the kidney’s unique role in handling lipid, the long-term exposure of the kidney to excess lipid burden is highly correlated with metabolic syndrome and an increased risk of developing renal cancer (17).

Micronutrients, such as vitamins and minerals, are essential for cellular homeostasis as cofactors for enzymatic reactions and as antioxidants. Overconsumption or under-consumption of certain micronutrients can compromise redox balance and the fidelity of DNA repair. Antioxidant vitamins, for instance, prevent reactive oxygen species from causing damage to cells and stabilize cell membranes, reducing mutational stress. Selenium, zinc and magnesium are essential minerals for maintaining genomic stability and enhancing detoxification pathways. To achieve a deeper functional understanding, recent metabolomic profiling of human renal cell carcinoma has demonstrated that the specific tissue of origin acts as the primary determinant for nutrient levels and metabolic flux, maintaining high localized concentrations of specific lipids and amino acids regardless of overall systemic variation (18).

A new and emerging area of investigation in nutritional oncology is phytochemicals, which are non-nutritive compounds present in plants. Phytochemicals, including polyphenols, flavonoids and carotenoids act as epigenetic regulators and can alter gene transcription through histone acetylation and DNA methylation. The capacity of these compounds to inhibit angiogenesis, induce apoptosis and modulate oxidative pathways classify them as among the most potent natural chemopreventive agents. The presence of these compounds in dietary patterns may explain why populations consuming large amounts of plant-based foods exhibit decreased rates of various cancers, although the strength of this association can vary, with the link to renal cancer being less pronounced than for malignancies such as colorectal cancer (18).

As a whole, groups of nutrients comprise an interconnected biochemical network that either maintains cellular homeostasis or destabilizes it. The interaction between carbohydrates, lipids, proteins and micronutrients determines metabolic response, hormone signaling and inflammation. Imbalance of nutrients causes an unbalanced state in the cellular environment, rendering renal cells susceptible to mutagenic and proliferative signals. Conversely, a number of nutrients such as fiber, antioxidants and bioactive phytochemicals appear to enhance metabolic resilience and slow the proliferation of cancer cells.

## 4 Dietary fiber and renal cancer risk reduction

Dietary fiber is the most influential component of the diet that regulates both immune functions and metabolism. For years it was thought that dietary fiber only had a beneficial effect on digestion, however, it is becoming increasingly apparent that it may also act as a biochemical regulator that will regulate oxidative status, inflammation and the initiation of cancerous processes. Its impact on renal cancer prevention is related to its capacity to alter metabolic pathways and systemic mediators that directly affect the health of renal cells. As a metabolic filter and an endocrine gland, the kidney is uniquely responsive to changes in these mediators. Dietary fiber consumption therefore indirectly but importantly, affects the molecular environment within which renal tumors grow and advance.

There is no single type of dietary fiber. Rather there are numerous types of indigestible components of plant material, including, cellulose, hemicellulose, pectins, beta-glucans, lignin, and resistant starch, etc. These types of fiber are digested in the upper gastrointestinal tract and then fermented in the large bowel by commensal microbiota. The fermentation process generates short chain fatty acids (SCFA’s) including acetate, propionate, and butyrate, which are signaling molecules that can regulate gene expression, cellular metabolism, and immune responses. Thus, through this biochemical pathway from the gut, dietary fiber exerts effects beyond the intestine and serves as a communication conduit between the gut and other organs including the kidneys (19).

Consumption of a high fiber diet results in the improvement of renal health by virtue of its anti-inflammatory effects. The SCFA’s generated from the fermentation of fiber interact with G-protein–coupled receptors on the surface of immune and epithelial cells resulting in the suppression of pro-inflammatory cytokine production including interleukin-6 and tumor necrosis factor-alpha. Chronic inflammation is a well-documented precursor to tumorigenesis since it establishes an environment rich in growth factors and oxidative intermediate products that contribute to DNA damage and the generation of mutations. While direct randomized controlled trials demonstrating that fiber prevents RCC incidence are lacking, a high-fiber diet is associated with improved systemic inflammatory and metabolic profiles (20). Through these mechanisms, increased fiber intake may contribute to an environment that is less conducive to oncogenic transformation in renal tissues.

High fiber intake also impacts the development of kidney cancer by altering oxidative stress. Due to its high metabolic rate, the kidney continuously produces reactive oxygen species (ROS) that can damage DNA, proteins, and lipids, leading to mutations and increasing the likelihood of developing cancer. The fermentation products of fiber, specifically butyrate, are known to activate antioxidant transcription factors in colorectal models (21). While direct evidence in human renal epithelium is still emerging, it is hypothesized that circulating SCFAs could exert similar cellular effects in the kidney, thereby creating an unfavorable environment for oxidative stress-induced carcinogenesis.

A second major mechanism of metabolic control through homeostasis is the ability to maintain a stable level of metabolism. Diets high in fiber improve the sensitivity of the pancreas to insulin and keep blood glucose levels at a relatively stable level; this will reduce the activation of insulin-like growth factor signaling (IGFS) which is very similar to signaling pathways involved in cell proliferation and tumor formation. Fiber also promotes fat degradation by sequestering bile acids and cholesterol in the small intestine and ultimately lowering the amount of fat in the body. Lower levels of fat stored in the body will decrease the lipotoxic stress that may lead to renal inflammation and fibrosis, both of which are common causes of increased likelihood of developing cancer (22).

The importance of the “gut-kidney axis” was recently identified as a new area of research concerning the relationship between the consumption of dietary fiber and kidney function. Products of the breakdown of fiber are absorbed into the circulatory system and can directly influence the functions of immune cells, endothelial cells, and the redox balance of the body. The short-chain fatty acids (SCFA) produced in the colon have been found to be epigenetic regulators of gene expression in renal tissues by affecting the acetylation status of histones, which in turn affects the expression of inflammatory and apoptotic genes. For example, butyrate has been shown to inhibit the activity of histone deacetylases that remove acetyl groups from histones, activating the expression of tumor suppressor genes and down-regulating the expression of pro-oncogene transcription factors. This is one of the many ways that dietary components can affect gene expression and cancer risk over time.

In addition to affecting epigenetic regulation, fiber supports the functional capacity of the detoxification systems of the liver and kidneys by increasing the excretion of potential carcinogens from the fecal stream and by reducing the reabsorption of uremic toxins in the bloodstream when the kidneys are not functioning properly. Diets high in fiber support the structural integrity of the normal flora of the colon and protect against the colonization of pathogenic bacteria that can produce toxic substances such as ammonia and nitrosamines. A healthy and balanced population of microorganisms in the colon protects the barrier function of the intestinal mucosa and reduces the systemic burden of toxins, thus protecting the renal tissue from the metabolic consequences of toxin exposure (23).

Epidemiological studies demonstrate that populations that consume diets high in fiber have lower rates of incidence for renal and other metabolic-related cancers than those who consume low-fiber diets. Although these epidemiological studies do not establish causality, they suggest a consistent association of physiologic benefit with nutrients rich in fiber. The biochemical, immunologic, and epigenetic mechanisms described above describe a unifying model that defines the role of fiber as a systemic regulator that maintains homeostasis and reduces carcinogenic stimuli in renal tissue (24).

When evaluating these beneficial effects, it is essential to distinguish between localized biological plausibility and proven clinical causality. Currently, the robust biochemical mechanisms underlying fiber metabolism are most clearly defined in experimental model systems and colorectal tissue research, whereas RCC-specific evidence in human populations remains primarily associative. While high fiber patterns facilitate improved metabolic status and reduced systemic inflammation, actions that potentially indicate a capacity for renal resilience, current clinical conclusions regarding their role in the direct prevention of renal carcinogenesis should be interpreted with appropriate caution. Therefore, rather than established causal tools, the discussed nutritional metabolites are categorized as promising molecular leads that necessitate further targeted longitudinal validation within renal oncology frameworks.

Dietary fiber serves as a biochemical sentinel, providing numerous protective effects by controlling metabolic signaling, maintaining immune equilibrium and enhancing the antioxidant defense systems. It also mediates the communication between the gut and kidneys via the gut-kidney axis, suggesting that it may represent a novel nutritional approach to prevent kidney cancer. The next chapter builds upon these mechanisms and reviews the molecular pathways used by fiber and its metabolites to interact with and potentially inhibit oncogenic signaling networks in renal cells (25).

### 4.1 Molecular pathways mediating fiber’s anti-cancer action

Dietary fiber’s molecular mechanisms for protecting against renal cancer are also very complex and involve an interlocking set of pathways that regulate energy homeostasis, inflammation, oxidative protection, and cell fate decisions. Fiber is also a biochemical intermediary between the gut microbiome and other organs which produces metabolites that can modulate gene expression and signal transduction. The evidence for these effects includes alterations in the AMPK/mTOR, NF-κB, and PI3K/Akt pathways, as well as the way SCFAs influence gene activity (26).

#### 4.1.1 AMPK/mTOR axis and metabolic regulation

The AMPK/mTOR pathway is one of the primary pathways that influence by the products of fiber-derived metabolism. AMPK is a cellular energy-sensing enzyme that is activated when the cell’s nutrient availability decreases and/or when the body is in a state of metabolic stress. SCFA (short-chain fatty acid), particularly butyrate and propionate activate AMPK through alterations in the AMP/ATP ratio and by activating upstream kinases such as LKB1. Once AMPK is activated it inhibits mTOR complex 1 (mTORC1); mTORC1 is a central regulator of cell growth, protein synthesis, and angiogenesis in kidney tumor cells (27). In this manner, the ingestion of dietary fiber indirectly suppresses the metabolic changes that typically occur to renal cells during oncogenic transformation through inhibition of anabolic processes that are typically stimulated at these times. mTOR signaling does not only inhibit the growth of tumor cells; it stimulates autophagy, which is the cellular process for degrading damaged organelles and proteins. Autophagy will be inhibited by the accumulation of oxidative damage debris in cells that appears to contribute to genomic instability and cancer (28).

#### 4.1.2 Inflammatory signaling and NF-κB modulation

The most common reason for an individual to develop kidney cancer is due to prolonged or chronic inflammation in their body. The fiber you consume, along with the fermentation products it produces, can act as anti-inflammatory agents to help control chronic inflammation by altering the functioning of the NF-κB pathway. This pathway is responsible for controlling the transcription of the genes involved in the processes that result in chronic inflammation and inhibit apoptosis. Butyrate will also inhibit histone deacetylase activity. Inhibiting histone deacetylase results in reduced expression of cytokines regulated by NF-κB, such as IL-6 and TNF-*α*. Furthermore, butyrate acts upon the G-protein-coupled receptors GPR41 and GPR43 to initiate anti-inflammatory signaling through the down-stream effectors of these receptors, ultimately leading to the inhibition of inflammatory transcription (28).

This modulation of inflammation will reduce the number of macrophages and neutrophils entering the renal tissue, therefore reducing the amount of cytokines they are exposed to on a chronic basis. Chronic cytokine exposure is one of the factors contributing to DNA damage and tumor promotion. The reduction of NF-κB activity is synergistic with AMPK signaling to inhibit angiogenesis and oxidative stress, thus canceling-out other cancer causing signals (29).

#### 4.1.3 Epigenetic remodeling and gene expression control

Gene expression is influenced by dietary fiber over an extended period through mechanisms of epigenetic modification. Epigenetic modification affects how genes are expressed, but does not modify the DNA sequence itself. A specific short chain fatty acid (SCFA), butyrate, inhibits histone deacetylase (HDAC) enzyme activity, thereby allowing for the relaxation of the chromatin structure to facilitate the transcription of tumor suppressor genes and silence oncogenes.

The process of epigenetically modifying or “reprogramming” cellular gene expression reactivates critical cellular pathways involved in apoptosis, DNA repair, and the generation of antioxidants. Importantly, this type of modification is reversible; thus, continued consumption of dietary fiber will maintain a gene expression profile that is continuously resistant to malignant transformations. In essence, fiber functions to rewire cellular memory through this method to ensure the maintenance of renal epithelial homeostasis and decrease the likelihood of carcinogenic reprogramming of the cell (30–32).

#### 4.1.4 Oxidative stress and mitochondrial homeostasis

The production of reactive oxygen species (ROS) acts as an intermediary signal molecule or mediator of genomic mutations. Overproduction of ROS can result in mutagenesis, lipid peroxidation, and dysfunctional proteins. Fermentation of fiber increases the body’s antioxidant potential through activation of the Nrf2 pathway, which is the principal regulatory element of cellular detoxification and redox homeostasis.

Butyrate separates Nrf2 from its inhibitor, Keap-1 allowing Nrf2 to translocate into the nucleus and induce the transcription of antioxidant enzyme coding genes such as glutathione peroxidase and catalase. The resultant increase in antioxidant activity decreases ROS levels thereby improving the integrity of mitochondrial membranes, reversing apoptosis-resistance characteristic of kidney cancer cell populations. Therefore, dietary fiber aids in maintaining healthy mitochondria; this is essential in maintaining metabolic homeostasis (33, 34).

#### 4.1.5 Gut–kidney axis and immune modulation

**Figure 3:**
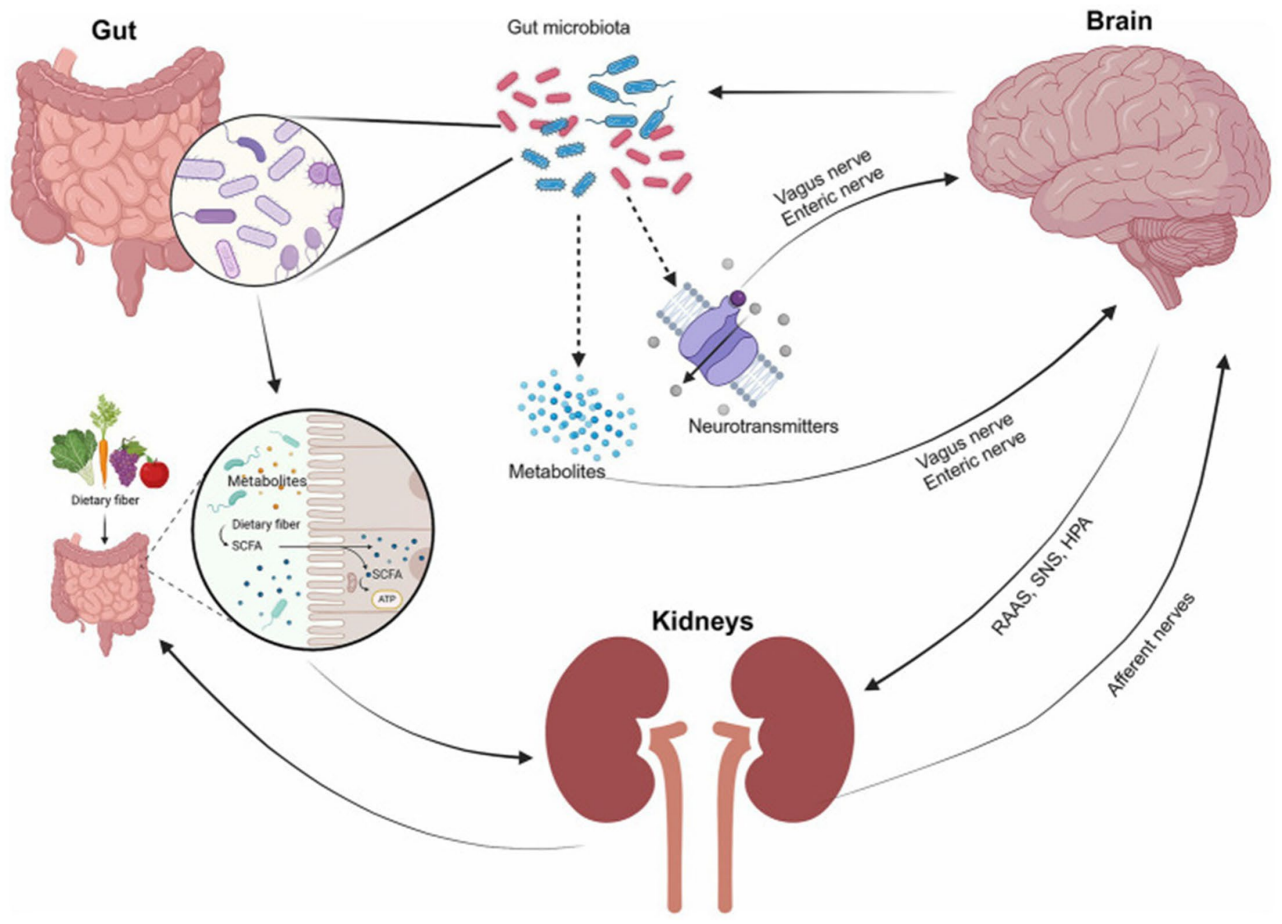
The interaction of the gut, brain, and kidneys. The gut microbiota serves as the key medium of communication between the gut, brain, and kidneys. The gut microbiota plays a crucial role in producing metabolites and neurotransmitters that impact brain function through interactions with the vagus nerve and the enteric nervous system. The gut also influences kidney function through metabolites, while the kidney interacts with the brain through hormonal pathways (e.g., RAAS, the SNS, the hypothalamic–pituitary–adrenal (HPA) axis, and afferent nerves) (92).

A significant two-way communication link between the Gut-Kidney Axis influences the function of the kidney’s immunological and metabolic processes through the action of metabolites produced by the gut microbiota. The production of SCFAs via fiber fermentation enables the immune system to modulate the type of immune response (i.e., anti-inflammatory) through the promotion of the differentiation of Regulatory T cells while inhibiting the generation of the pro-inflammatory Th17 cell response thereby eliminating the inflammation associated with tumor growth in the kidney. In addition to immune modulation, SCFAs enhance the endothelial function within the kidney through increased production of Nitric Oxide thereby enhancing renal blood flow and reducing localized hypoxia, which is a well-known initiator of the HIF pathway that generates new blood vessels necessary for tumor growth and metastasis. Overall, fiber consumption creates a micro-environment that is less conducive to tumor growth and metastasis by altering systemic as well as localized immune responses (35, 36). Figure 3 illustrates the complex communication network between the gut, brain, and kidneys, highlighting the central role of gut microbiota.

## 5 Other key nutrients in renal carcinogenesis

While dietary fiber is an important nutritional component for prevention, it is ultimately the total nutrient composition of a given individual’s diet that will determine how probable cancer development of the kidney will be at both the physiological and molecular level. The biochemical properties of a wide variety of nutrients are known to provide both protective as well as facilitatory effects toward renal carcinogenesis depending on the biochemical properties of the nutrients themselves, the amounts ingested and the extent of assimilation into metabolism. Understanding these nutrients further provides us with knowledge of how dietary factors may alter the biological process of cancer at the molecular level.

### 5.1 Antioxidants and oxidative defense

Oxidative stress is one of the main reasons why people develop kidney cancer; because when the body has too many reactive oxygen species (ROS) and not enough antioxidant defenses, DNA can be damaged, lipids can become oxidized, and proteins can become misfolded. The molecular antioxidants (vitamins C & E, carotenoids, etc.) will help reduce oxidative stress by neutralizing free radicals to restore redox balance. Water soluble vitamin C acts as a reducing agent in the cytoplasmic and extra cellular space to stop the formation of ROS. Vitamin E works to prevent the propagation of lipid peroxidation chain reactions that would destroy cellular membranes. Selenium is also necessary as a co-factor for glutathione peroxidase, an enzyme that breaks down hydrogen peroxide. As a result, antioxidants such as these have been shown to decrease mutagenic stress, resulting in fewer mutations occurring in renal epithelial cells, leading to a lower incidence of oncogenic mutations (37–39).

### 5.2 Polyphenols and phytochemicals

The anti-inflammatory and anti-proliferative activities exhibited by polyphenol compounds extracted from a variety of sources, including tea, fruits, and vegetables, impact multiple pathways associated with cancers. Curcumin, resveratrol, and catechin are three polyphenolic compounds that exhibit anti-inflammatory and anti-proliferative activity in addition to blocking transcription factors such as NF-κB and STAT3, inhibiting cyclooxygenases and altering apoptosis-regulated proteins, and affecting epigenetic marks that can activate tumor-suppressing genes that have been silenced. In renal cancer models, these phytochemicals have demonstrated the capability to inhibit angiogenesis through reduced VEGF signaling and disruption of HIF stabilization. The combination of the antioxidant and anti-inflammatory activity of polyphenols with the activity of dietary fiber creates an unfavorable cellular environment for the development of cancer. Moreover, the ability of polyphenols to modulate gut microbiota results in the increased synthesis of beneficial metabolites, creating an indirect relationship to the gut-kidney axis established through fiber fermentation (18).

### 5.3 Micronutrients and trace elements

Zinc, Magnesium & Iron—as micronutrients, are essential to support both enzymatic activity and maintaining Genomic Integrity. As a component of multiple DNA Repair Enzymes, including several Transcription Factors; when zinc levels fall, p53’s ability to regulate cell cycle arrest is impaired, and cells become more susceptible to oxidative damage. Magnesium is involved in the energy dependent enzymatic reactions (ATP dependent) and assists in ensuring DNA Replication Accuracy. Although Iron is required for Hemoglobin formation, excess amounts of iron will lead to increased risk of developing Cancer due to an accelerated rate of the Fenton Reaction creating highly Reactive Hydroxyl Radicals. Therefore, maintaining a Balance of these micronutrients rather than simply Adequacy is critical to establishing the Renal Cell Resilience (40).

Calcium and Vitamin D also play key roles in regulating renal cancer. Through its Receptor-Mediated Signaling, Vitamin D induces cell cycle arrest and Apoptosis in numerous Epithelial Malignancies. Additionally, Vitamin D’s Immunomodulatory Effects decrease the secretion of Pro-Inflammatory Cytokines which have been shown to slow the proliferation of Kidney Cancer. While excessive Calcium Supplementation, without Physiological Regulation, increases the risk by altering how the kidneys handle calcium and increasing oxidative damage (41).

### 5.4 Harmful dietary components and oncogenic acceleration

A number of nutrients and forms of diet can cause significant amounts of metabolic and inflammatory stress that may lead to increased risk of renal cancer. High protein diets, particularly diets rich in red or processed meats produce excess nitrogenous wastes and lipid peroxides that place large amounts of metabolic burden on the kidney’s ability to filter the blood. Elevated sodium consumption increases blood pressure and generates reactive oxygen species at the level of the vascular endothelium thus impairing kidney function. Diets with excessive amounts of saturated fat activate lipid mediated signaling pathways such as PPAR and SREBP, which are involved in promoting lipogenesis and oxidative inflammation. This combination of factors creates an environment conducive to DNA damage, chronic hypoxic conditions, and alterations in metabolic states associated with the promotion of tumor development (42–44).

These combined effects of dietary stressors enhance kidney vulnerability through the additive effects of metabolic overload, mitochondrial dysfunction, and long term inflammatory signaling; therefore, renal carcinogenesis should be viewed as a systemic consequence of sustained nutritional imbalance rather than a single molecular event (see Table 1).

**Table 1:** Nutrient and compound profiles: sources, biological effects, and molecular mechanisms in renal cancer context.

Nutrient/compound	Primary source	Biological effect on renal tissue	Key molecular pathways influenced	Mechanistic outcome
Dietary Fiber (Soluble & Insoluble) (25)	Whole grains, fruits, vegetables, legumes	Enhances metabolic stability, reduces inflammation, supports microbiome diversity	AMPK/mTOR, NF-κB, HIF-1α, Nrf2	↓ Cell proliferation, ↓ angiogenesis, ↑ apoptosis, ↑ antioxidant defense
Short-Chain Fatty Acids (Butyrate, Propionate, Acetate) (26)	Fermentation of dietary fiber in gut	Acts as epigenetic modulator and energy substrate for colonocytes; regulates immune tone	HDAC inhibition, GPR41/43 signaling	↓ Inflammatory cytokines, ↑ tumor suppressor gene activation
Antioxidants (Vitamin C, E, Selenium, Carotenoids) (39)	Citrus fruits, nuts, seeds, green vegetables	Neutralizes reactive oxygen species (ROS), maintains redox balance	Nrf2/Keap1, MAPK, p53 stabilization	↓ DNA damage, ↑ cellular detoxification, ↑ genomic stability
Polyphenols (Resveratrol, Curcumin, Catechins) (93)	Green tea, berries, turmeric, grapes	Anti-inflammatory and anti-angiogenic; modulates gene expression	NF-κB, STAT3, VEGF, HIF pathways	↓ Angiogenesis, ↓ invasion, ↑ apoptosis, ↑ tumor suppression
Micronutrients (Zinc, Magnesium, Selenium) (41)	Whole grains, seafood, leafy greens	Supports DNA repair and enzymatic antioxidant systems	p53, DNA repair enzymes, GPx, SOD	↑ Genomic integrity, ↓ oxidative injury

## 6 Emerging perspectives in nutrigenomic and metabolomic integration

The human body operates as a highly connected biochemical network in which nutrients, metabolites, and signaling molecules interact and change rapidly across multiple organ systems. Through the lens of systems biology, the understanding of these connections for renal cancer provides important insights about how different types of food affect the operation of biological pathways and ultimately how that affects the outcome of the disease. Instead of functioning independently, nutrients are part of a network of regulatory processes that determine the operation of the genome, the rate of metabolic flow, and immune signaling (45).

At the center of this integrative paradigm is nutrigenomics (the study of how nutrients affect the genes), which is an important part of understanding the integrated effects of nutrients on biological processes. As a ligand, co-factor and/or epigenetic regulator, nutrients activate/inhibit specific gene networks through a variety of mechanisms including but not limited to transcriptional activation of receptors, post-translational modification of enzymes and reorganization of chromatin through epigenetic means. The complexity of the regulatory networks involved in renal cancer are further complicated by the sensitivity of the kidney to oxidative and metabolic stresses. Nutrients alter the operation of many signaling pathways (e.g., PI3K/Akt, AMPK/mTOR, HIF) that help regulate the balance between cell homeostasis and cancer promotion.

Metabolomics, an essential element of systems biology, allows researchers to map the changes in metabolism caused by the consumption of food. Metabolomic profiling of circulating metabolites provide a way to understand the effects of nutrient consumption on tumor metabolism, oxidative stress and immune modulation. For example, diets rich in fiber increase the levels of short-chain fatty acids (SCFA), which in turn activates AMPK and improves mitochondrial function. Conversely, diets rich in fat causes an accumulation of acylcarnitine, which increases oxidative stress and inflammation. When metabolomic data is combined with gene expression data, we get a complete picture of how dietary changes operate at a molecular level to change renal tumor physiology (46).

The ability of single nutrient classes to influence oncogenesis is often amplified through pathway cross-talk. For example, SCFAs derived from fiber fermentation do not merely activate AMPK; their influence extends to inhibitother core pathways such as the PI3K/Akt and Wnt/*β*-catenin cascades, thus interferingwith cell proliferation and stemness (36, 37). Similar crosstalk is observed where butyrate diminishes HIF-1α stabilization, thereby limiting angiogenesis (38). Concurrently, antioxidants can indirectly temper HIF-1α signaling by reducing the intracellular ROS required for its stabilization. This complex interplay, where nutrients modulate multiple nodes like mTOR, NF-kB, and HIF simultaneously, demonstrates a systems-level impact on renal cell biology that transcends single-target interventions (47).

Systems-level approaches using network modeling and computational simulation have begun to define patterns of interaction among the elements of these systems that reveal how variations in the composition of diet affect renal carcinogenesis. These models illustrate that nutrients act as regulatory nodes that direct metabolic pathways toward either promoting or suppressing tumor growth. While the development of fully personalized dietary advice from multi-omics data remains a goal for future research, recent advances provide foundational insights. For example, metabolomic studies in RCC have identified that specific circulating lipids, such as acylcarnitines, are correlated not only with tumor aggressiveness but also with dietary fat intake. Such findings suggest that profiling an individual’s metabolome could 1 day help stratify patients who might benefit most from targeted dietary interventions aimed at lipid metabolism modulation. This approach, though still investigational, highlights a viable pathway toward integrating nutrigenomics into RCC management.

In addition, immune regulation emerges as an important integrative axis in this network. Nutrients like fiber, polyphenols and omega-3 fatty acids modulate immune checkpoints by influencing the release of cytokines, antigen presentation and T-cell differentiation. These immunometabolic changes transform the tumor microenvironment from one that suppresses the immune response to one that enhances immune surveillance.

## 7 Translational and clinical implications of nutrient modulation in renal cancer

Understanding how diet contributes to renal cancer at the biochemical level (how it influences the progression of renal cell carcinoma) supports the idea that nutritional factors should transition from being considered as secondary or even passive background contributors to cancer development to being recognized as primary drivers in all aspects of kidney cancer development, treatment and survivorship. There is evidence of direct interaction between the bioactive compounds in diets and many key cellular mechanisms involved in both the formation of renal cell carcinoma and the body’s response to energy status, such as the VHL/HIF pathway, the PI3K/AKT/mTOR pathway, NF-kappa B, AMPK, reactive oxygen species generation/neutralization, and DNA methylation/demethylation processes (48–52). This understanding has been supported by numerous studies using experimental animal models of renal cell carcinoma and by a growing number of epidemiologic studies of renal cell carcinoma incidence among humans. The translation of this scientific understanding into clinical practice will require the identification of how populations’ eating habits affect their risk for developing renal cell carcinoma, the use of food/nutrient consumption to identify which groups of people are at the greatest risk, and the design of clinical programs that include nutritional interventions to prevent recurrence and improve survival.

## 8 Epidemiological and dietary pattern evidence in renal cancer

Large cohort studies and case–control studies show a correlation between overall dietary patterns and renal cell carcinoma (RCC) incidence (53–55). Instead of studying individual nutrients, studies look at how people eat as a whole, including “Western”-style diets high in red/processed meats, saturated fats, refined grains & sugary beverages, and “prudent,” plant-forward, high-fiber diets rich in fruits, vegetables, legumes, whole grains, and unsaturated fats. Energy-dense Westernized diets are associated with obesity, insulin resistance, hypertension, and low-grade chronic inflammation—all established risk factors for RCC (14, 16, 56–58). Conversely, plant-dense diets high in phytochemicals and fiber are often associated with lower RCC risk and/or better metabolic profiles in at-risk individuals.

Mechanistically, these epidemiologic data are consistent with the nutrient-pathway interactions described earlier. Saturated fat & simple sugar diets promote adiposity, hyperinsulinemia, and dyslipidemia—generating an endocrine milieu that activates PI3K/AKT/mTOR signaling and increases proliferative and anti-apoptotic pathways in renal cells (59–62). Excessive sodium intake may also increase hypertension and intraglomerular pressure, further damaging renal parenchyma and making it more susceptible to carcinogenic stressors (63–67). Conversely, low intake of fiber, antioxidants, & micronutrients can impair endogenous defenses against oxidative stress and DNA damage, leaving renal tissue vulnerable to mutations in tumor suppressor genes and other metabolic genes (68–72). These perturbations can act synergistically with genetic predispositions such as germline or somatic alterations in VHL or other genes related to renal cancer to accelerate RCC initiation and progression.

Conversely, dietary patterns emphasizing whole plant foods provide higher levels of soluble and insoluble fiber, complex carbohydrates with a lower glycemic load, and polyphenol rich vegetables/fruits, which contribute to improved insulin sensitivity, reduced inflammatory tone, and better redox balance (73–75). The production of short chain fatty acids in the colon from increased fiber intake supports activation of AMPK and enhanced mitochondrial function through immune modulation along the gut-kidney axis (76, 77). Antioxidant and micronutrient sufficiency provides for enhanced genomic stability through improved DNA repair and more efficient detoxification of reactive species. Therefore, epidemiologic data showing a decrease in incidence of metabolic disorders and certain cancers in populations consuming higher amounts of Mediterranean-like or similar plant-forward diets can be viewed indirectly in the context of RCC as evidence that such diets buffer key oncogenic pathways active in RCC (78).

Consistent with these mechanisms, global prospective datasets provide a quantified understanding of the molecular resilience offered by nutrition. To provide a rigorous clinical context, specific quantitative associations gathered from pooled analyses must be highlighted. Observational evidence suggests that high adherence to fiber-rich and plant-dense diets is associated with an RCC Risk Reduction (RR) typically ranging from 0.82 to 0.90 (95% CI: 0.75–0.93 for fiber) across diverse Caucasian and East Asian populations. In distinct contrast, classical clinical risk factors exert higher relative weights: hypertension is consistently reported with an RR between 1.6 and 2.6 (95% CI: 1.5–2.8), while obesity demonstrates RRs of 1.6–1.82 (95% CI, 1.55–2.0) (54). These data emphasize that while nutrient patterns stabilize metabolic circuitry, they function within a hierarchy of standard risk factors, serving as an adjunctive rather than singular preventative intervention. Table 2 provides a direct synthesis of these evidentiary magnitudes and confidence intervals.

**Table 2:** Comparison of relative risk estimates for modifiable RCC risk factors synthesized from primary cohort and meta-analytical evidence.

Category	High-modifiable factor	Pooled risk estimate (RR, 95% CI)	Evidence quality (GRADE)	Population consistency
Nutritional	High Dietary Fiber and Veg	0.82–0.90 (95%CI: 0.75–0.94)	Strong	Consistent globally
Nutritional	Excess Ultra-processed Sugar	1.15–1.21 (95%CI: 1.02–1.33)	Moderate	Population-dependent
Clinical	Chronic Hypertension	1.61–2.61 (95%CI: 1.50–2.85)	Strong	Extremely High
Metabolic	High BMI (>30 kg/m^2^)	1.61–1.82 (95%CI: 1.55–2.10)	Strong	Extremely High
Lifestyle	Active Tobacco Use	1.30–1.52 (95%CI: 1.25–1.77)	Strong	High

Furthermore, current clinical cohorts acknowledge that while nutrient signals bolster cellular integrity, diet-based associations are subject to systemic recall bias. This necessitates the integration of these findings into a multimodality prevention plan that accounts for individual uremic risk and genomic susceptibility.

However, there are many important limitations in the current body of evidence. Large cohort studies frequently assess diet using food frequency questionnaires or recall tools that are subject to misclassification and recall bias. Residual confounding by lifestyle variables associated with diet, such as physical activity, smoking, and socioeconomic status, is difficult to account for even after adjusting for multiple variables. In some settings, relatively small numbers of RCC cases limit statistical power to detect effects of specific dietary patterns. Definitions of diet scores differ between studies, and the duration of follow-up also varies. Despite these constraints, mechanistic data in concert with trends in epidemiology support the concept that diet composition, particularly fiber density, lipid quality, and overall plant-to-animal ratio of foods, influences RCC risk at the population level.

## 9 Nutrient-based prevention and risk stratification framework

Nutrient intake can be thought of as an area of modifiable risk for renal carcinogenesis that can be assessed and addressed in both public and clinical health practices; given the known mechanisms of how diet relates to the pathogenesis of renal carcinoma and the demonstrated associations of diet with renal carcinoma occurrence, there is a clear basis to pursue the assessment of the risk of renal carcinoma associated with nutrient intake. Individuals at increased risk of developing renal carcinoma (including obese individuals, individuals with metabolic syndrome, hypertensive individuals with a long-standing history of hypertension, individuals with chronic kidney disease, and individuals with a first-degree relative with renal carcinoma) typically have similar patterns of metabolic dysfunction, oxidative stress, and chronic low grade inflammation (79, 80). These intermediate phenotypes are also the areas where nutrient intake can affect them. Therefore, the goal of a nutrient-based prevention strategy would be to shift the molecular environment of individuals at highest risk away from oncogenic signals and towards metabolic adaptation.

A nutrient-based prevention framework for renal cancer centers on the synergistic action of dietary fiber, bioactive phytochemicals, and essential micronutrients. Instead of focusing on single mechanisms, this approach leverages the combined capacity of these components to restore metabolic homeostasis, mitigate chronic inflammation, and protect genomic integrity in renal epithelial cells. High-fiber diets contribute to improved insulin sensitivity and generate SCFAs, which possess epigenetic and anti-proliferative properties discussed earlier. Complementing this, antioxidants and phytochemicals fortify cellular defenses against oxidative DNA damage, while sufficient intake of micronutrients ensures the fidelity of DNA repair enzymes. Together, these elements form a multi-pronged nutritional strategy aimed at reducing the cumulative molecular burden that drives renal carcinogenesis (81–84).

Therefore, a feasible prevention approach could involve the development of composite dietary indexes that assess the combined effects of these nutrients and not individual components in isolation. The indexes could weigh high consumption of whole grains, legumes, vegetables, fruits, and unsalted nuts positively and processed meats, refined carbohydrates, sugar-sweetened beverages, and food products that contain high levels of saturated fats and sodium negatively. Ultimately, individuals could be stratified into two categories based on their dietary index score (high vs. low) and receive recommendations tailored to their level of risk over time. Emerging disciplines such as nutrigenomics and metabolomics may allow even more precise stratification of nutritional-related risk, in that nutritional-related risk is assessed in the context of molecular markers such as circulating lipid profiles, inflammatory cytokine levels, oxidative stress biomarkers, and metabolites that are uniquely associated with renal carcinoma.

Despite the significant potential for utilizing multi-omics to define personalized prevention, several hurdles remain to be cleared for the transition toward localized precision nutrition. Currently, the most significant challenges involve the lack of longitudinal Randomized Controlled Trials (RCTs) specifically focused on RCC-intervened dietary cohorts and the reliance on retrospective epidemiological records, which are vulnerable to subjective bias. Furthermore, the lack of validated surrogate biomarkers that link nutrient intake to direct intra-tumoral molecular outcomes—such as changes in metabolic flux—limits the ability to move from associative population data to validated causation. Therefore, until these biosignatures are robustly quantified through prospective clinical trials, nutritionally-informed strategies should remain integrated as adjunctive components within established renal oncology guidelines rather than standalone interventions.

Operationalizing this intervention also involves practical challenges. Nutri-tional behaviors are influenced by cultural, economic, and personal factors, and maintaining long term compliance is challenging unless individuals are supported. Furthermore, patients with specialized renal conditions, such as those with decreased reserve, must balance potassium-rich fruits and phosphorus-intense foods to avoid excessive metabolic burdens. For these reasons, a nutrient-based prevention approach should be considered an evidence-informed, biologically plausible, and relatively low-risk intervention that provides an additional tool for clinicians and public health officials to utilize in conjunction with well-established interventions such as smoking cessation, blood pressure management, weight management, and elimination of occupational exposures.

### 9.1 Integrating nutritional strategies into renal cancer treatment and survivorship

Nutrient modulation has implications for the entirety of renal cancer care—from prevention through the survivorship phase—and is particularly important in patients receiving systemic treatments (e.g., tyrosine kinase inhibitors, immune checkpoint inhibitors) who have less functional renal tissue upon which to rely for metabolism and excretion (14, 85, 86).

The integration of diet into the treatment plan for patients with renal cell carcinoma (RCC) will be based on the premise that nutritional strategies are supportive of the intended therapeutic goals of oncologic therapies; thus, diets that are rich in whole plants, moderate in quality protein, and low in sodium and processed sugar can support stabilization of blood pressure, reduction of systemic inflammation, and maintenance of an optimal body composition.

Sufficient yet not excessive protein intake during the perioperative period will support wound repair and immune system competency. In addition, attention to sodium and fluid intake will minimize the risk of developing hypertension or edema secondary to therapies that target angiogenic signaling pathways (87, 88).

It is critical to monitor renal function, electrolytes, and nutritional status in patients receiving systemic therapies, especially those with a decline in glomerular filtration rate or preexisting chronic kidney disease. Patients who require modifications to their fiber-rich, plant-forward diets to avoid excessive potassium or phosphorus burdens benefit from collaboration between oncologists, nephrologists, and renal dietitians to develop customized meal plans that modify the amount and type of protein consumed, control mineral intake, and provide adequate caloric and micronutrient supplies.

For some patients, supplementation with specific nutrients, including omega-3 fatty acids or certain antioxidants, may be beneficial; however, the use of high-dose antioxidant supplements requires caution, as they may blunt the oxidative mechanism by which many chemotherapeutic agents induce cytotoxic effects.

Once the acute treatment phase is complete, nutrition becomes a long-term tool to support patient well-being, decrease recurrence risk, and manage comorbid conditions associated with decreased nephron mass, such as cardiovascular disease, metabolic syndrome, and chronic kidney disease.

Survivors of RCC are typically at increased risk of cardiovascular disease, metabolic syndrome, and chronic kidney disease due to shared risk factors, decreased nephron mass, or therapy-related sequelae (89–91). The adoption of dietary patterns that include high intakes of fiber, plant-based fats, and unrefined carbohydrates while limiting sodium and added sugars may help lower blood pressure, improve lipid profiles, and promote weight loss, which indirectly protects the kidney and reduces systemic conditions that may contribute to tumor recurrence.

Survivors of RCC must be supported in their efforts to adopt sustainable dietary habits that fit their personal preferences, financial constraints, and lifestyle, as abruptly prescribed or overly restrictive dietary recommendations will not be adopted and maintained over the long term.

Table 3 illustrates the relationship between nutrient-focused strategies, molecular targets, and clinical stages of care for patients with renal cancer. It provides a summary of the multiple nutrient-focused strategies that may be employed to support the health of patients with renal cancer, the predominant nutrient characteristics of each strategy, the underlying biological pathways and processes influenced by each strategy, the clinical phases of the renal cancer continuum in which each strategy is most applicable, and the clinical considerations and potential precautions that must be addressed when employing each strategy.

**Table 3:** Nutrient-focused strategies across the renal cancer continuum: mechanistic rationale and clinical application (94–96).

Nutritional strategy or pattern	Dominant nutrient features	Primary mechanistic targets and processes	clinical stage of application	Key considerations and precautions
Plant-forward, high-fiber dietary pattern	Emphasis on whole grains, legumes, vegetables, fruits, nuts; low intake of processed meat and refined sugars	Modulation of insulin and IGF signaling; AMPK activation; attenuation of PI3K/Akt/mtor and NF-κb; improved oxidative balance and gut–kidney axis signaling	Primary and secondary prevention; long-term survivorship	Requires adjustment in advanced CKD to avoid hyperkalemia and excessive phosphorus; adherence facilitated by culturally appropriate food choices
Antioxidant- and polyphenol-rich intake	High consumption of colorful fruits and vegetables, teas, herbs, and spices containing vitamins C and E, carotenoids, and polyphenols	Enhancement of Nrf2-mediated antioxidant responses; stabilization of p53; modulation of angiogenic and inflammatory signaling including VEGF and NF-κb	Prevention; adjunct to treatment; survivorship	High-dose supplements should be used cautiously during certain chemotherapies; whole-food sources generally preferred for safety and synergy
Controlled-protein, low-sodium plan in reduced renal reserve	Moderate total protein with emphasis on plant and high-biologic-value sources; limited sodium and processed foods	Reduction of intraglomerular hypertension; mitigation of uremic toxin production; decreased RAAS activation and vascular stress	Post-nephrectomy, chronic kidney disease, treatment-related nephrotoxicity	Protein and electrolyte targets must be individualized based on GFR, body mass, and catabolic state; close monitoring by nephrology and dietetics teams is required
Limitation of processed meat, saturated fat, and added sugars	Restriction of cured and smoked meats, deep-fried foods, high-fat dairy, confectionery, and sugar-sweetened beverages	Decrease in lipid peroxidation and oxidative stress; reduction in obesity-related inflammation and metabolic overload that amplify oncogenic signaling	Primary prevention; risk reduction in high-risk individuals; survivorship	Behavioral support may be needed to maintain changes; potential benefits extend to cardiovascular and metabolic outcomes beyond renal cancer

## 10 Conclusion

An understanding of the role of nutrients in modulating cancer development has transformed nutritional science into an integral part of oncologic management rather than just an adjunctive tool. The application of this knowledge in the context of renal cell carcinoma represents a major advance in terms of the prevention and reduction of risk of developing renal cell carcinoma, as well as providing an additional therapeutic option. Additionally, using nutrients to modulate biological processes provides a safe, affordable, and evidence-based method to provide complementary support to current medical treatment options.

As a preventive measure, the use of dietary fiber and its bioactive components to regulate oncogenic pathways indicates that nutritional intervention may play a significant role in primary cancer prevention. Encouraging individuals to consume diets rich in fiber, plant-based foods, and antioxidants may assist in reducing chronic inflammation and metabolic stress, both of which are key factors in the development of renal carcinogenesis. Therefore, these recommendations align with broader public health initiatives designed to address metabolic disorders, hypertension, and obesity, which share common pathophysiological mechanisms with renal tumorigenesis.

Additionally, these results provide support for the integration of nutritional oncology into standard patient management protocols. Modulation of diet may improve responsiveness to treatment and reduce adverse effects of treatment (i.e., toxicity), therefore supporting the use of targeted therapies and immunotherapy agents. For example, nutrients that activate AMPK or inhibit mTOR may be used to enhance the efficacy of pharmacological inhibitors of analogous pathways. Similarly, antioxidants and anti-inflammatory compounds may assist in protecting kidney function from the oxidative injury caused by chemotherapy or radiation therapy.

One of the greatest challenges in translating these concepts into practice is the development of dietary recommendations tailored to each individual’s genetic profile, metabolic status, and microbiome composition. Recent advances in the fields of nutrigenomics and metabolomics now enable the customization of treatment based on molecular information, thus opening the possibility of precision nutrition in cancer care. Such personalized approaches may elucidate how effectively individuals utilize specific nutrients, whether they are deficient or over-supplemented, and develop dietary plans to suppress tumor growth while maintaining homeostasis of metabolic systems.

Finally, nutritional counseling may be particularly relevant for individuals who have completed cancer treatment. Survivors of cancer often experience alterations in their metabolic systems and suffer from fatigue and immune dysfunction. A diet high in fiber, antioxidants, and plant-based protein may help restore balance to the body’s systems and decrease the likelihood of recurrence of cancer.

In order for clinical oncologists to incorporate dietary research into their treatment strategies, there needs to be collaboration between molecular biologists, physicians, and nutritionists to design studies to evaluate the efficacy of dietary interventions in renal cancer patients. Additionally, randomized controlled clinical trials to evaluate the impact of nutrient-pathway modulation in renal cancer patients will be critical in validating laboratory findings and establishing evidence-based dietary guidelines.
